# The excitable nature of polymerizing actin and the Belousov-Zhabotinsky reaction

**DOI:** 10.3389/fcell.2023.1287420

**Published:** 2023-10-31

**Authors:** Michael Riedl, Michael Sixt

**Affiliations:** Institute of Science and Technology Austria (ISTA), Klosterneuburg, Austria

**Keywords:** actin polymerization, Belousov-Zhabotinsky reaction, excitable systems, chemical waves, chemical oscillators, cell migration, coupling, synchronization

## Abstract

The intricate regulatory processes behind actin polymerization play a crucial role in cellular biology, including essential mechanisms such as cell migration or cell division. However, the self-organizing principles governing actin polymerization are still poorly understood. In this perspective article, we compare the Belousov-Zhabotinsky (BZ) reaction, a classic and well understood chemical oscillator known for its self-organizing spatiotemporal dynamics, with the excitable dynamics of polymerizing actin. While the BZ reaction originates from the domain of inorganic chemistry, it shares remarkable similarities with actin polymerization, including the characteristic propagating waves, which are influenced by geometry and external fields, and the emergent collective behavior. Starting with a general description of emerging patterns, we elaborate on single droplets or cell-level dynamics, the influence of geometric confinements and conclude with collective interactions. Comparing these two systems sheds light on the universal nature of self-organization principles in both living and inanimate systems.

## 1 Introduction

From the collective pulsations of heart cells to the rhythmic flickering of a firefly, the dynamics exhibited by excitable systems are a captivating phenomenon. Excitable systems respond to external stimuli or internal fluctuations with propagating waves of activity. Once coupled together, multiple excitable units—such as coupled cells in a tissue—can show population-level activity patterns and produce robust higher-level self-organization in the absence of a central orchestrator. Understanding emergent behavior requires a solid comprehension of the dynamics at all levels of the system.

The polymerization reaction of actin exhibits excitable dynamics and gives rise to waves that travel along the cellular membrane (Gerisch et al., 2004; Allard and Mogilner, 2013; Devreotes et al., 2017; Inagaki and Katsuno, 2017; Gerisch et al., 2019; Beta et al., 2023). The emergent wave-like patterns result from the continuous and localized polymerization and depolymerization of actin (Vicker et al., 1997; Ruthel and Banker, 1998; Ruthel and Banker, 1998; Stankevicins et al., 2020; Zhan et al., 2020; Weiner et al., 2007; Riedl et al., 2023; Barnhart et al., 2011). Although the specific function of these actin waves is not well understood, they do interplay with various cellular activities and signal transduction processes and their dynamics is controlled by complex cascades of various regulatory proteins (Giannone et al., 2004; Weiner et al., 2007; Westendorf et al., 2013; Devreotes et al., 2017; Miao et al., 2019).

In this perspective article, we aim to convey an intuitive understanding of the excitable dynamics exhibited by polymerizing actin within cells. For this purpose, we will draw parallels between the waves of actin polymerization and a well understood system, displaying a remarkably similar behavior: the Belousov-Zhabotinsky (BZ) reaction. The BZ reaction is a well-studied example of a chemical oscillator that displays excitable dynamics (Zaikin and Zhabotinsky, 1970). This inorganic reaction produces self-sustained chemical oscillations, visible as propagating waves of periodically alternating color changes (Epstein and Pojman, 1998; Mikhailov and Ertl, 2017). It has served as a fertile toy model for nonlinear dynamical system theories and its investigation has provided essential contributions to our understanding of the principles of self-organization, reaction-diffusion dynamics, and the emergence of complex spatiotemporal patterns.

We will start with a sequential overview of both systems, focusing on the characteristic observable wave patterns and the mechanisms responsible for their emergence. Subsequently, we will compare analogue functionalities between the two systems. This includes self-propelled motion, the role of geometric constraints and the influence of external fields. Finally, we will explore collective effects such as synchronization.

It is worth mentioning that chemical oscillators and excitable systems are not strictly equivalent. Excitable systems show all or nothing responses to stimuli and require a threshold to be exceeded for activation. Chemical oscillators display sustained and mostly continuous oscillations in concentrations of reactants or products due to autocatalytic or self-regulating reactions. To discriminate between them based on observables is delicate, as it requires a detailed understanding of the underlying mechanisms that give rise to rather similar characteristics. These nuances are not central to the intent and purpose of this article and we will use the terms interchangeably.

## 2 Complex dynamics and emergent patterns of excitable waves

### 2.1 The Belousov-Zhabotinsky reaction

What we perceive as travelling waves are propagating chemical reactions. Their formation and propagation involve a complex series of chemical reactions. In the BZ system, these reactions rely solely on five key reagents: sulfuric acid, sodium bromate, sodium bromite, melanonic acid, and a metal ion catalyst such as ferroin. Melanonic acid acts as an organic substrate and is consumed during the reaction (Jahnke and Winfree, 1991). Ferroin acts both as a catalyst and an indicator, changing its oxidation state during the reaction, which results in the characteristic oscillating color changes between red (reduced form) and blue (oxidized form) (Cassani et al., 2021). The overall reaction results from over 80 elementary chemical steps (Gyorgyi et al., 1990). The five ingredients form a five-dimensional parameter space, mapping the emergent patterns based on the possible combinations of initial concentrations. On top of that, reaction dynamics can be heavily impacted by environmental factors such as temperature (Carballido-Landeira and Epstein, 2010; Howell et al., 2021) or externally applied fields (Agladze and De Kepper, 1992; Blank and Soo, 2003; Back et al., 2023), or by the spatial dimensions of the reaction container (Toth et al., 1994; Steinbock and Müller, 1998).

**FIGURE 1:**
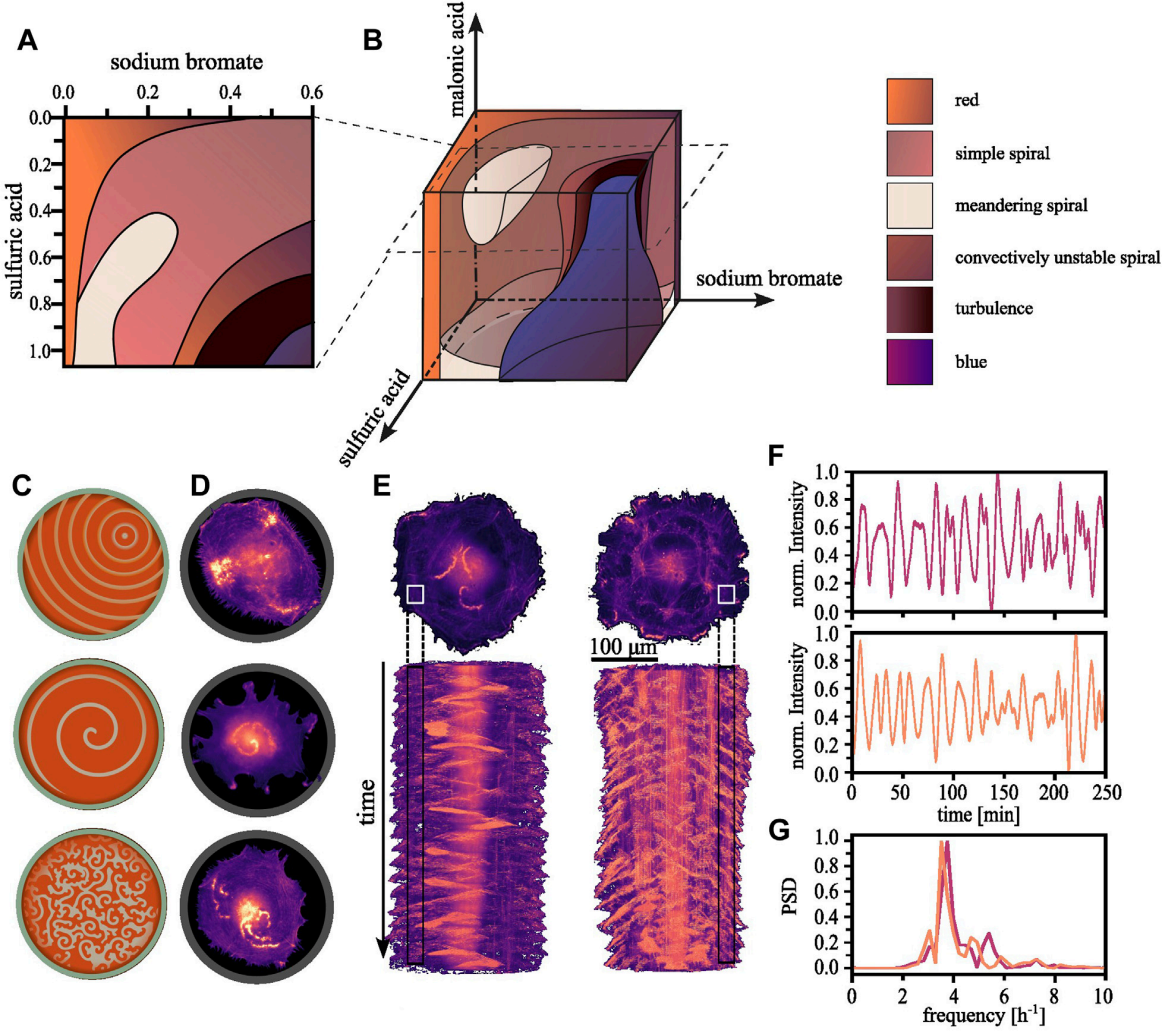
Regularity in the observable patterns. **(A)** By consecutively varying one of two parameters a two-dimensional section through phase space can be constructed. Each parameter combination gives rise to an emerging pattern which can be characterized, resulting into areas of with similar dynamics within the plane. **(B)** Subsequently, multiple such planes can be reconstructed into a three-dimensional representation [The here shown phase space diagram were reproduced with permission from Belmonte et al. (1997)]. **(C)** Schematically shown, the typical observable patterns for the BZ reactions range from concentrical or eccentrical waves, spiral waves and more complex or chaotic regimes. **(D)** We have found the equivalent patterns in aortic endothelial cells confined within adhesive circular confinements (D = 100 μm). **(E)** Long time sequences reveal that the random appearing wave dynamics are in fact periodic. Top: Single cells expanding onto a whole ring pattern showcasing the periodicity of the dynamics of the polymerizing actin waves (D = 200 μm, s = 30 μm). Bottom: The corresponding space-time projections. **(F)** The normalized localized actin intensity over time extracted from the regions indicated in. **(E)** Top corresponds to the left and bottom to the right cell. **(G)** The corresponding power density spectrum highlights the periodicity of ∼4 h^−1^.

While the basic BZ reaction kinetics are fairly well-understood, the system’s non-linearity and potential for exhibiting chaotic behaviour renders it sensitive to initial conditions and minor deviations can result in unproportioned changes in the outcome. The dynamics are deterministic and it is possible to map all outcomes to the initial conditions chosen. For a fixed concentration, multiple states can exist, a property termed bistability or multistability. Some of these states are less stable than others. The system’s history determines the eventual observed state, meaning its initial condition, and the manner in which control parameters are varied to reach that state. This phenomenon is known as hysteresis (Epstein and Pojman, 1998). It is informative to create phase space diagrams where one parameter is varied at a time while the others are held constant (Li et al., 1996; Belmonte et al., 1997; Zhou and Qi, 2001). In Figure 1A, we exemplify such a phase space diagram for the BZ reaction created by Belmonte and colleagues, who assembled multiple two-dimensional sections into a three-dimensional representation (Belmonte et al., 1997). When exploring the three-dimensional phase space diagram shown in Figure 1B, we find it populated by regions of various spiral states, which are bordered by the stationary, globally reduced or oxidized states. Though the characteristic concentric ring patterns depicted in Figure 1C (top) are absent under the referenced experimental conditions, the simple spiral state, exemplified in Figure 1C (center), fills most of the volume. Spirals arise from anisotropic condition in the medium, leading to a wavefront that curls onto itself (Kapral and Showalter, 1995). For other concentrations, chaotic or turbulent patterns can emerge, as displayed in Figure 1C (bottom). The front, bulk, and rear of the wave represent regions dominated by different stages of the cyclic reactions (Field et al., 1972):

1) At the wavefront, the initial set of chemical reactants—bromate, bromide, and the organic substrate such as malonic acid—undergo several autocatalytic and redox reactions. Ferroin, initially in its reduced (red) state, facilitates the oxidation of malonic acid by bromate. As the catalyst is oxidized, it changes to a blue color, marking the transition to the front of the propagating wave. 2) The bulk of the wave is characterized by the continued reactions of the generated intermediates. The bromine species continue their reactions with the other reactants. This leads to a dynamically changing but continuously high state of intermediate concentration. The bulk of the wave maintains the blue color indicative of the oxidized state of the catalyst. 3) At the rear of the wave, representing the final stage of the cycle, the system transitions to the recovery or refractory phase. Here, the residual bromine species are reduced back to bromide ions by the now reduced form of the ferroin catalyst, thereby restoring the original red color. Consequently, the concentrations of reactants are mostly, but not completely, restored, priming the system for the onset of a new wave.

### 2.2 Polymerizing waves of actin

Actin polymerization and the BZ reaction represent two strikingly different systems, one a biochemical polymerization and the other an inorganic reaction, yet they share commonalities, e.g., in the wave-like propagation of the reaction. In the BZ reaction, a mixture of chemicals exhibits oscillations in color as the reaction proceeds. In a similar cyclic fashion, actin polymerizes within cells and also this process, fundamental to numerous cellular functions, exhibits wave-like patterns.

Emergent patterns have been reported for a range of cell types (Inagaki and Katsuno, 2017; Beta et al., 2023), yet we observed them most prominently in single endothelial cells restricted within adhesive circular confinements at a diameter of 100 µm (Riedl et al., 2023). Actin polymerization within these cells displays the typical excitable behavior: traveling waves traversing the cell [Figure 1D (top)]. These waves interact with the plasma membrane. When approaching their vicinity, they push it outward. Upon colliding with another wave, neither can sustain itself within the recovering medium left in their wake, and as a result, both waves annihilate. Spiraling waves can also be observed as shown in Figure 1D (center) in addition to other more complex—and potentially turbulent—patterns with an increased frequency of nucleated waves [Figure 1D (bottom)] (Tan et al., 2020). Whether the different patterns serve specific cellular functions and how they are involved in biological processes such as cell motility and division, is still unclear.

Despite the seemingly random dynamics, actin polymerization follows an inherent order (see Figure 1E). The nucleation of new waves can be quantified and exhibits periodic oscillations (see Figure 1F) with a defined frequency over long timescales (see Figure 1G). However, individual cells within a population naturally vary in their intrinsic chemical composition. The instantaneous reaction dynamics depend on the dynamically deforming membrane, which acts as a geometric confinement. The interplay of these factors renders the experimental construction of a phase space diagram an enormous challenge. Nevertheless, such an imaginary phase space diagram of polymerizing actin might bear some resemblance to the BZ reaction diagram.

Much like the waves in the BZ reaction, waves of actin polymerization follow a conceptually similar triad of initiation, propagation, and termination, orchestrated by a complex network of molecular interactions (Devreotes et al., 2017; Miao et al., 2019).

1) The wave initiation, forming the wavefront of polymerizing actin, is prompted by the localized activation of nucleation promoting factors such as Wiskott–Aldrich syndrome proteins (WASP) and the WAVE regulatory complex (Weiner et al., 2007). These nucleation promoting factors, when activated by Rho family GTPases, regulate actin nucleating factors such as the actin-related protein 2/3 (Arp2/3) complex. This regulation triggers the polymerization of actin monomers into actin filaments, thereby marking the start of the wave. 2) As the wave progresses, the actin filamentous network density in the bulk of the wave increases due to sustained actin polymerization, facilitated by the activated Arp2/3 complex. Concurrently, actin depolymerizing factors, initiate the disassembly of actin filaments into actin monomers. These disassembled monomers can then be recycled and contribute to the ongoing polymerization. 3) Wave termination begins as actin filaments are extensively depolymerized into actin monomers, signifying the wave rear. During this final stage of the actin wave, signaling proteins, which previously activated the WASP and WAVE complexes, become inactive. This inactivation, paired with large-scale actin depolymerization, resets the system, thereby priming it for the emergence of a new wave.

## 3 From observation to functional insights

### 3.1 Movement in cells and droplets

Both systems demonstrate analogous spatiotemporal dynamics, a commonality that is reflected in another: Both can propel their containing vessel, but through different mechanisms.

**FIGURE 2:**
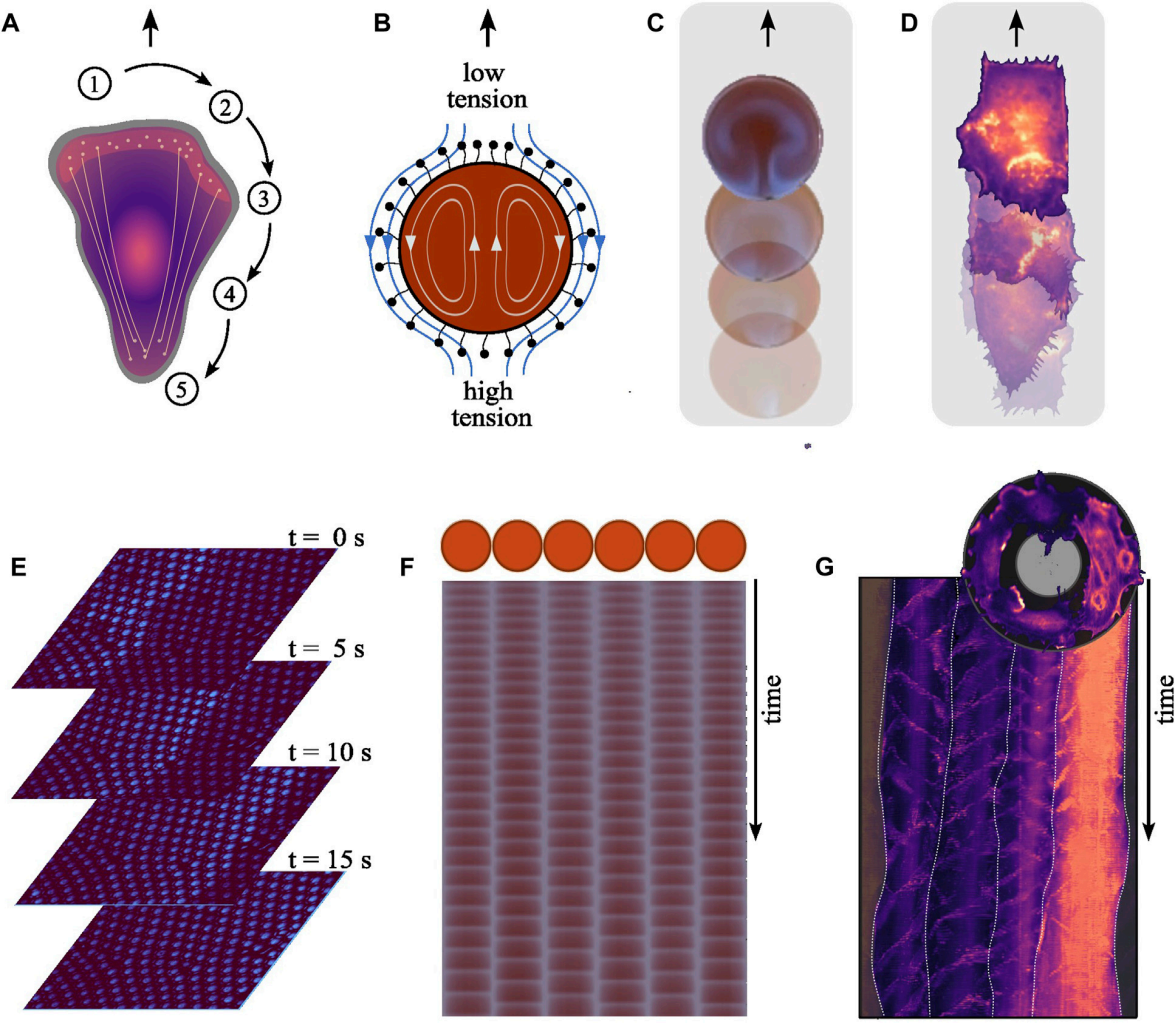
Mechanistic parallels between the systems. **(A)** Cell migration involves several spatiotemporally coordinated steps. (1) Upon polarization, the cell establishes a front-rear polarity, redistributing components to the newly formed front while others remain at the rear. (2) At the front, polymerizing actin pushes against the cell membrane forming protrusions, namely, lamellipodia and filopodia. (3) These new protrusions are anchored to the substrate through adhesions, inhibiting their retraction. (4) Subsequently, myosin motors generate pulling forces contracting the cell body, (5) which in turn triggers the retraction of the cell rear. Effective locomotion depends on the precise spatiotemporal coordination of these dynamics. **(B)** The oscillating chemical reaction inside a BZ droplet results in local periodic alternation in the concentration of reaction intermediates, including surfactants on the droplets surface modifying its tension. This gradient in surface tension across the droplet’s surface leads to flows along the interface, causing an equilibrating fluid motion from areas of lower surface tension (higher surfactant concentration) to areas of high surface tension (and lower surfactant concentration). The propulsive force generated by the flow acting on the surrounding liquid displaces the droplet, resulting in its self-propelling behavior. **(C)** Droplets encompassing the BZ reaction exhibit self-propulsion, advancing forward with each periodically emerging wave [Reproduced with permission (Back et al., 2023)]. **(D)** We recently showed the positive correlation between the nucleation frequency of polymerizing actin waves and the speed of aortic endothelial cells migrating on adhesive line patterns. (Width = 50 μm). **(E)** Traveling wave form across communicating oscillator droplets encapsulated within bilayer membrane. Each droplet exhibits a diameter of 30 μm. The time interval between shown images is 5 s [Created from original data provided by the authors (Thutupalli and Herminghaus, 2013)]. **(F)** Schematic of an array showcasing six oscillating droplets surrounded by a lipid membrane within an emulsion system and from the time series derived space-time plot for each droplet [Reproduced with permission (Torbensen et al., 2017a)]. **(G)** Top: An image illustrating the collective migration of aortic endothelial cells along a ring-shaped adhesive pattern (diameter = 300 μm, width = 35 μm). Bottom: The polar-transformed space-time representation of the time-series data of a configuration of four cells on a ring during collective rotation. The frequency-locked state is apparent due to the uniform vertical spacing between the periodically nucleating actin polymerization waves, both within individual cells and across the population. White lines indicate the boundaries between individual cells.

Cell migration involves a cyclical sequence of spatiotemporally coordinated biochemical reactions, described in Figure 2A (Seetharaman and Etienne-Manneville, 2020). In essence, upon polarization, coordinated actin and myosin activities at the cell’s front and rear, respectively, facilitate protrusions and cell body contraction, enabling effective movement.

When encapsulated within droplets, the BZ reaction can cause self-propelled motion reminiscent of cell migration (Kitahata et al., 2002; Suematsu et al., 2016; Back et al., 2023). This movement is driven by Marangoni flows, which arise driven by gradients in surface tension along the interface between the internal solution and the surrounding oil, as illustrated in Figure 2B. This results in flows both within and around the droplet, latter propelling the droplet forward as depicted in Figure 2C (Yoshikawa et al., 1993; Kitahata et al., 2002; Thutupalli et al., 2011).

In both systems, microscopic processes translate into self-propelling macroscopic motion. In migrating cells, the nucleation frequency of actin waves correlates positively with the migration speed (see Figure 2D) (Riedl et al., 2023). How actin wave dynamics mechanistically relate to the cyclic sequence driving cellular locomotion is still unclear. Whether actin waves cause surface tension driven flows—alike those moving a BZ droplet—remains also to be seen. Current research focuses on whether and how tension is propagated along the plasma membrane (Shi et al., 2018; Cohen and Zheng, 2020; De Belly et al., 2023). Another question is how individual players in the machinery of cell migration feedback on the wave dynamics. For example, cell migration generally uses adhesions that are functionalized as linkages to the substrate. The strength of these adhesion alters the frequency of the internal actin waves, showcasing the influence of the exterior on the internal reaction kinetics (Barnhart et al., 2017).

### 3.2 The role of geometric confinement

Geometric confinements alter the characteristic timescales between the diffusive transport and reaction of the involved chemicals (Toiya et al., 2008). In the BZ reaction, this limitation affects the emergent patterns and the corresponding wave frequencies, to the point of preventing waves from forming altogether or pushing the system into a binary state, switching between the globally oxidized or reduced states (Toth et al., 1994; Epstein and Pojman, 1998; Steinbock and Müller, 1998; Thutupalli and Herminghaus, 2013). A droplet, encapsulating the reacting solution, functions as the geometric confinement (Toiya et al., 2008). Lipids stabilize such droplets and similar to the plasma membranes of cells, they can act as an active component participating in the reaction dynamics. When deforming or plastically changing their volume by incorporating additional lipids, the membrane of BZ droplets can alter the dynamics of the chemical reaction it contains (Szymanski et al., 2013).

Confinements also influence the emergent wave patterns in cells. We found the cleanest examples of polymerizing actin waves in endothelial cells confined on circular adhesive patterns with a diameter of 100 μm (Riedl et al., 2023). Endothelial cells naturally attempt to cover the totality of the provided area. Thereby, they transition into a nearly two-dimensional shape. We speculate that the confinement diameter together with the resulting two-dimensionality—in a similar manner to the BZ reaction—shifts the phase space into a regime that preferentially gives rise to persistent wave patterns (Toth et al., 1994). This size dependent behavior of polymerizing actin has been also observed in fused *D. discoideum* cells (Yochelis et al., 2022). Upon cell size increase, actin not only forms the commonly observed broad ring-shaped wave segments but also exhibits sharp planar traveling pulses, which travel at increased speeds. However, there is still a need for an experimental study to systematically explore how cell size affects the behavior of polymerizing actin.

Hence, the exploration of geometric constraints could provide a common ground for comparison of these two systems. It might contribute to a more profound understanding of how spatial boundaries, either in a chemical setting or in living cells, can govern self-organizing patterns and behaviors. Especially in the biological context it is unclear how the ratio between cytoplasm and cell membrane, acting as a platform for actin polymerization, influences the emergent waves. Geometric confinements can further introduce curvature to the cell membrane. Locally altering the reaction conditions by changing the ratio between reaction and diffusion timescales.

### 3.3 The influence of external fields

Cells respond to various gradients in external fields such as chemical, mechanical, and electrical fields, and can even generate their own (Insall et al., 2022; Alanko et al., 2023). In the context of cell migration, the pursuit of these gradients is referred to as taxis. Electrotaxis, the ability of cells to follow electrical fields, can be observed in both droplets and cells (Zajdel et al., 2020; Back et al., 2023). While the underlying mechanism in cells is not fully understood, the propagation direction of polymerizing actin waves also align with an applied electrical field (Yang et al., 2022). A plausible hypothesis links the directional bias in cell migration to the polarization of membrane-bound proteins (Allen et al., 2013; Kobylkevich et al., 2018). Whether this theory can be extended by including actin waves as a mediator between polarization and migration remains to be seen.

In the context of the BZ reaction, the presence of an external electric field acts on the charged molecules within the solution, altering the emergent spatiotemporal patterns. An applied electric field triggers symmetry breaking and influences the speed of the emergent waves. Either accelerating or decelerating them depending on their propagation direction relative to the direction of the field (Agladze and De Kepper, 1992; Back et al., 2023). An applied electric field biases the motion and direction of a BZ droplet. This results from the combination of the aforementioned effects and a spatial shift of the wave nucleation point with respect to the droplet center. Observing such a shift of the nucleation point of actin waves concomitantly with the application of an electric field would be an interesting find.

### 3.4 The emergence of collective behavior

When BZ droplets are placed in proximity to each other, they can exhibit synchronized oscillations (Toiya et al., 2008; Thutupalli and Herminghaus, 2013; Totz et al., 2018). Here, synchronization result from the interaction between droplets through the diffusion of molecules involved in the BZ reaction into the shared surrounding medium (Delgado et al., 2011). This process establishes a chemical coupling between droplets (Figure 2E), allowing them to communicate and synchronize their internal chemical oscillations (Figure 2F). The coupling strength can be tuned by changing the composition of the droplets’ membrane (Thutupalli and Herminghaus, 2013; Torbensen et al., 2017a). Whether the aforementioned surface tension driven flows also act as a coupling mechanism is unclear. Thus, even though each droplet has its own inherent oscillatory behavior, the coupling leads to collective behavior up to the scale of a communication network (Torbensen et al., 2017b; Chang et al., 2018).

Cells can synchronize their behavior through various forms of coupling, including direct contact (e.g., via gap junctions) or through diffusible signaling molecules. In the context of polymerizing actin waves, it is unclear exactly which coupling mechanisms give rise to synchronization. We recently showed that when cells move collectively, the nucleation of actin waves can be synchronized across multiple cells, setting a uniform collective speed (Figure 2G) (Riedl et al., 2023). This feedback provides a mechanism for cells to communicate and coordinate their activities, much like the chemical communication that occurs between BZ droplets.

In both systems, synchronization can lead to complex spatiotemporal patterns and coordinated behavior on scales larger than the individual units. While the coupling in the BZ droplet system is believed to be purely chemical and dictated by diffusion, the coupling in multi-cellular systems is more complex, involving potentially both biochemical and mechanical aspects. Understanding these processes presents an exciting challenge and could shed light on fundamental principles of self-organization and synchronization in biological and chemical systems.

## 4 Conclusion

Predicting the emergent dynamics of a population of self-organizing, autonomous units based on the properties of the individuals is currently an elusive task. Yet, nature consistently demonstrates an innate ability to self-organize, forming living organisms. To develop a conceptual understanding and a predictive framework, it is crucial to understand the behavior of the individual units that serve as building blocks, along with their interactions. By highlighting the similarities in the spatiotemporal dynamics of such minimal units in the form of cells polymerizing actin and droplets containing the BZ reaction, we hope to have conveyed the universality within the spatiotemporal behavior of excitable systems. The inorganic reaction, with its simpler composition and well-characterized kinetics, offers an accessible and adjustable system to explore. Meanwhile, actin polymerization offers a biological perspective and ties findings to a functional output such as cell migration. The insights gained from studying the BZ reaction may offer a stepping stone to address the questions that linger, especially in the dynamics of actin.

## Data Availability

The raw data supporting the conclusion of this article will be made available by the authors, without undue reservation.
